# Tree_RNA-Align: RNA Secondary Structure Clustering and Classification Based on Tree-Structure Alignment

**DOI:** 10.3390/ijms27167327

**Published:** 2026-08-17

**Authors:** Zhijie He, Chengzhen Xu, Xiaomin Wu

**Affiliations:** 1 School of Computer Science and Technology, Huaibei Normal University, Huaibei 235000, China; 13231028001@163.com; 2 College of Life Sciences, Huaibei Normal University, Huaibei 235000, China

**Keywords:** Tree_RNA-Align, cluster analysis, RNA secondary structure, RNA classification, tree-structure alignment

## Abstract

Clustering and classification of RNA secondary structures are central to understanding RNA function. However, widely used alignment methods, such as LocARNA and bpRNA-align, are not explicitly designed to exploit the hierarchical relationships among RNA structural elements, limiting their applicability to complex, multi-branched structures. In this study, we introduce Tree_RNA-Align, a novel method for RNA secondary structure clustering and classification based on a tree-structure alignment algorithm. The method transforms dot–bracket structures into tree representations, in which multibranch loops and stems serve as nodes, thereby preserving the hierarchical relationships among structural elements. It integrates a bottom–up hierarchical comparison for clustering with a top–down comparison for classification and prediction. Notably, classification experiments on five RNA families (16S rRNA, group_I_intron, RNase P, SRP, and tmRNA) achieved a micro-averaged *F*1-score of 0.933 (range across families: 0.760–0.981) and effectively identified representative structures within each family. Overall, these results suggest that incorporating classification can further improve RNA secondary structure prediction, demonstrating the utility of Tree_RNA-Align for structural analysis and functional annotation.

## 1. Introduction

RNA is an essential biological macromolecule that performs fundamental functions, including genetic information transmission and protein synthesis, and plays a central role in key cellular processes, such as gene expression regulation, catalytic reactions, and viral replication [1,2,3]. Similar to proteins, RNA function is closely related to its spatial structure [4], and its secondary and tertiary structural conservation is considerably greater than that of its primary sequence. Therefore, the systematic analysis of RNA structural characteristics and the relationship between its structure and function has become a major focus of molecular and computational biology [5,6].

Advances in experimental technologies, including X-ray crystallography, cryo-electron microscopy, and nuclear magnetic resonance, together with the widespread adoption of high-throughput structural detection methods, have led to a significant increase in the number of RNA structures recorded in databases such as the Protein Data Bank (PDB) in recent years. Since 2010, more than 50% of the independently identified RNA structures available in the PDB have been solved [7]. Moreover, high-throughput structure-probing technologies have generated experimentally supported structural data for more than 150 unique transcriptomes [8], providing a substantial foundation for systematic RNA structural analysis. However, this rapid accumulation of structural data has created major challenges in extracting meaningful information from large and complex RNA structure datasets, identifying hidden structural similarities through structural comparison, uncovering structural patterns via clustering and classification, inferring the functions of uncharacterized RNAs, and discovering novel structures. Nonetheless, this accumulation of data has driven continuous innovation in RNA structure comparison, clustering, and classification algorithms and tools.

Non-coding RNA (ncRNA) plays critical regulatory roles in cell proliferation, differentiation, apoptosis, and disease-related processes, and its function is highly dependent on its spatial structure [9]. As structural similarity often reflects functional homology [10], RNA structural alignment, clustering, and classification have emerged as core methods for analyzing ncRNA function and exploring the principles governing RNA evolution. Traditional RNA research often focuses on sequence analysis; however, due to high sequence variability [11], even sequences with extremely low similarity can form similar spatial structures and perform similar functions [12]. Therefore, functional inference based solely on sequence analysis is challenging, necessitating a shift from sequence- to structure-based analysis. Furthermore, conventional structural alignment is primarily based on sequence-similarity extension, making it poorly suited for sequences with less than 60–70% similarity. Although specialized tools, such as bpRNA-align [13] and LocARNA [14,15], improve alignment accuracy through optimized gap-penalty strategies and structure-specific substitution matrices, they remain limited when aligning complex and long RNA structures. Therefore, despite their usefulness, existing RNA structural alignment methods require further optimization to improve the alignment of long RNAs and structurally flexible RNAs and to support the rapid processing of large datasets.

Traditional RNA structural clustering relies primarily on structural alignment. Clustering is typically performed by calculating structural similarity matrices and grouping them using classical clustering algorithms, such as *k*-means and hierarchical clustering, or specialized tools, such as GraphClust [16,17], RNAScClust [18], and GINClus [19]. Clustering performance has been improved by integrating multiple data sources, incorporating structural conservation information, and applying graph isomorphism networks alongside clustering algorithms. For example, GINClus achieves over 87.88% accuracy in clustering known ring motifs. However, existing methods generally do not handle large datasets well and exhibit poor clustering performance for dynamically conforming RNAs. Furthermore, RNA classification is heavily dependent on structural-feature extraction and machine learning algorithms, with most classification models developed only for specific RNAs, such as 12S rRNA [20]. Consequently, the absence of universal standards and systems for RNA classification makes it difficult to identify new RNA structures and meet the growing demand for RNA structural classification. Moreover, studies often view the comparison, clustering, and classification of RNA as separate tasks [21,22,23], limiting the development of an integrated framework that improves both efficiency and accuracy.

To address these gaps, we present Tree_RNA-Align, a novel method for RNA secondary structure clustering and classification based on tree-structure alignment. This method addresses the limitations of existing tools through the integrated optimization of RNA structural alignment, clustering, and classification strategies. Overall, this study provides a robust framework for supporting future research on RNA structure and function.

## 2. Results

### 2.1. Cluster Analysis

**Figure 1:**
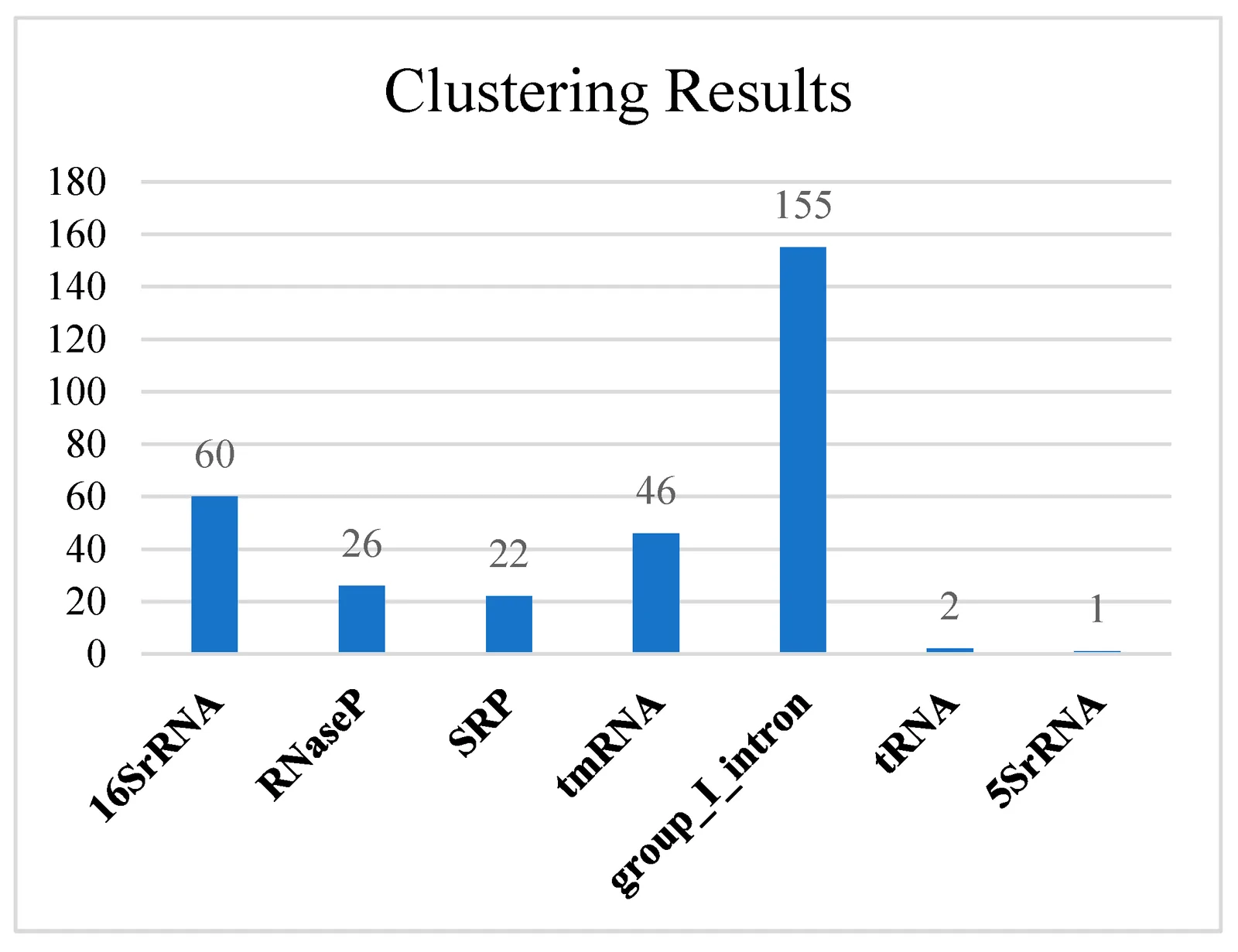
RNA secondary structure clustering results.

Figure 1 presents the number of categories obtained for each of the seven families through clustering. The group_I_intron family yielded the largest number of clusters, which may reflect either the intrinsic structural diversity within this family, the wide range of sequence lengths among its members, or both (Figure 1). Although the clustering algorithm imposes sequence-length constraints, as presented in Equation (1), the overall secondary structure remains consistent. The 16S rRNA, RNase P, SRP, and tmRNA families contain comparatively fewer clusters, indicating that they share several identical substructures that differ only slightly in sequence length. The 5S rRNA and tRNA families contain the fewest clusters, indicating minimal variation in sequence length and reflecting secondary structures that conform to a fixed template.

Based on these clustering results, we selected the five categories containing the most structures and the most visually distinct features in each family to examine their characteristics and proportions. Using the structures with the most representative tree representations and the highest prevalence within each category as templates, we summarized the structural characteristics of each category.

### 2.2. 16S rRNA

**Figure 2:**
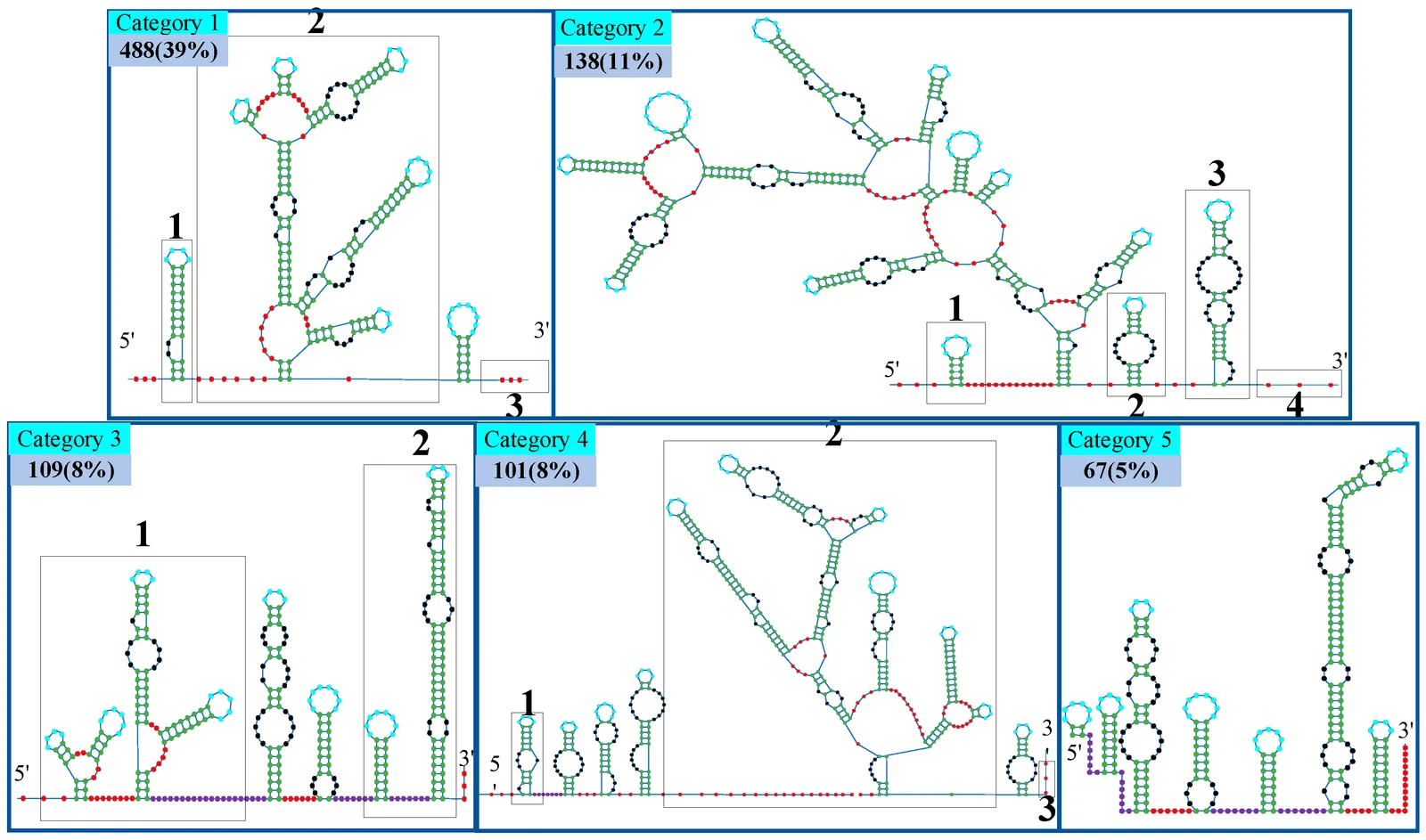
RNA secondary structures in the five categories with the most sequences in the 16S rRNA family, as well as their quantities and proportions. The numbers in blue represent the number and proportion of structures; black, inner or convex ring; blue, hairpin ring; red, multi-branched loop or free base; green, stem area; purple, continuous unpaired sequence of at least 15 bases; unmarked, the current structure (similar below).

Based on our clustering analysis, the top five categories containing the most structures accounted for 71% of all structures within the 16S rRNA family (Figure 2). In Category 1, some RNAs lack the stem region at position 1, while free bases at position 3 generate additional stem regions without multi-branch loops, depending on RNA length (Figure 2). Additionally, Category 1 features multibranch loops at position 2. In more complex RNAs, the free bases on either side pair to form a multibranch loop with the stem region on the right, within which additional stem regions form. In Category 2, although the RNAs share several common stem regions, most contain a stem region with a multibranch loop, differ in sequence length, and typically feature additional stem regions at position 4. In Category 3, most structures possess two stem regions at position 1, where free bases on either side may further form a multibranch loop and a stem region on the left; position 2 may contain fewer or more stem regions, depending on sequence length. In Category 4, owing to variation in sequence length, positions 1 and 3 may contain more or fewer stem regions, and the first multibranch loop at position 2 may open and transform into free bases, reflecting the complexity of the RNA secondary structure. In Category 5, most RNAs conform to the structure illustrated in Figure 2.

### 2.3. RNase P

**Figure 3:**
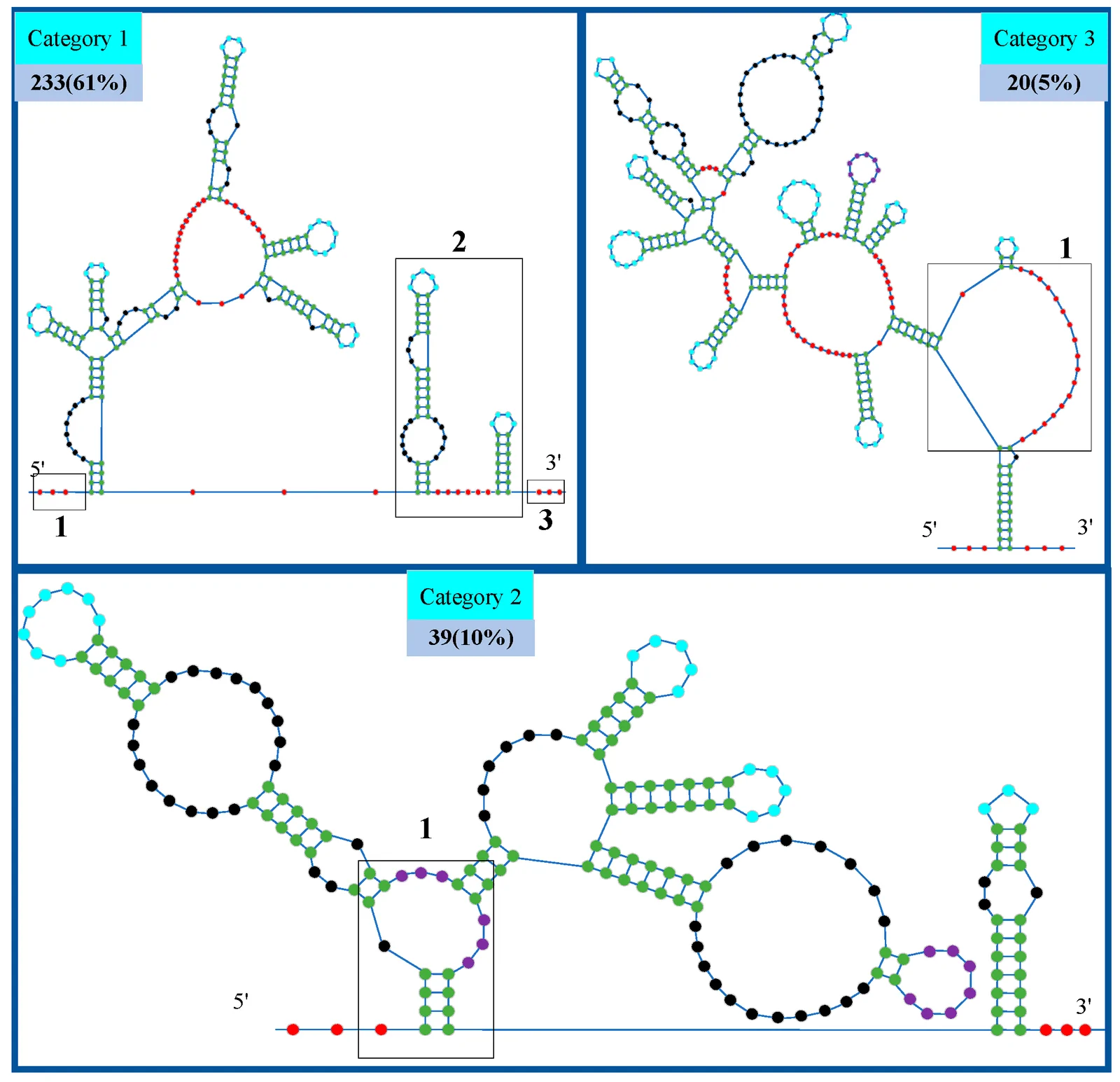
RNA secondary structures in the three categories with the most structures in the RNase P family, as well as their quantities and proportions. The numbers in blue represent the number and proportion of structures; black, inner or convex ring; blue, hairpin ring; red, multi-branched loop or free base; green, stem area; purple, continuous unpaired sequence of at least 15 bases; unmarked, the current structure.

Based on our clustering analysis, the top three categories accounted for 76% of all structures within the RNase P family (Figure 3). In Category 1, position 1 may contain a stem region, while two stem regions at position 2 may further form multibranch loops on either side, and position 3 may feature multiple stem regions, depending on differences in RNA sequence length (Figure 3). In Category 2, the multibranch loop at position 1 may open to form free bases. Similarly, in Category 3, the multibranch loop at position 1 likewise opens to form free bases.

### 2.4. SRP

**Figure 4:**
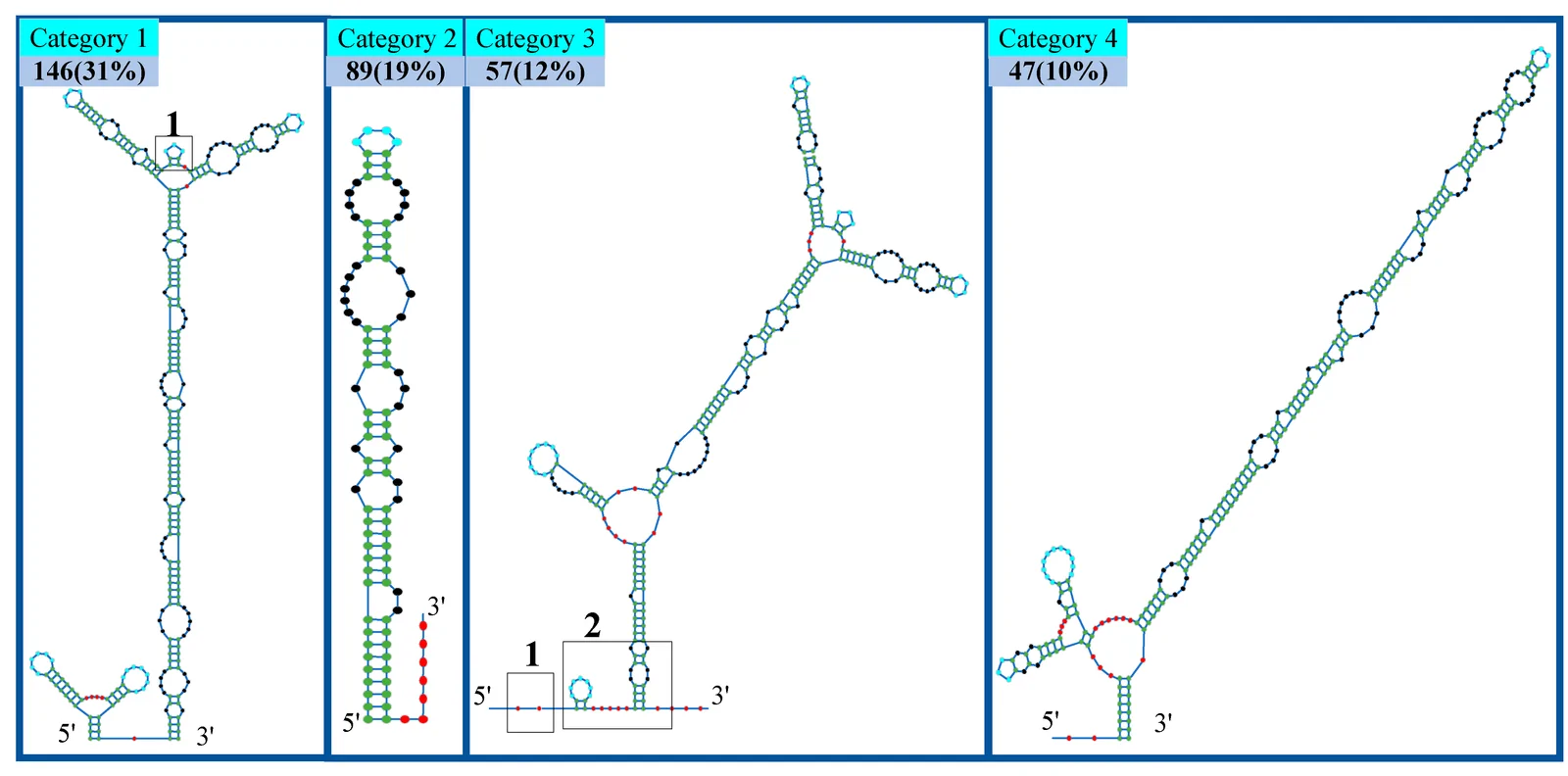
RNA secondary structures in the five categories with the most structures in the SRP family, as well as their quantities and proportions. The numbers in blue represent the number and proportion of structures; black, inner or convex ring; blue, hairpin ring; red, multi-branched loop or free base; green, stem area; unmarked, the current structure.

In the SRP family, the four categories with the most structures, accounting for the greatest structural diversity, represented 72% of all structures (Figure 4). Categories 1, 2, and 4 exhibit essentially the same structures shown in Figure 4, without forming more complex secondary structures. Rather than being illustrated separately, Categories 3 and 5 are described relative to this representative structure: Category 5 is derived from Category 1 by deleting the stem region at position 1, whereas Category 3 shares the overall architecture of Category 1 but includes an additional stem-loop configuration at positions 1–2.

### 2.5. tmRNA

**Figure 5:**
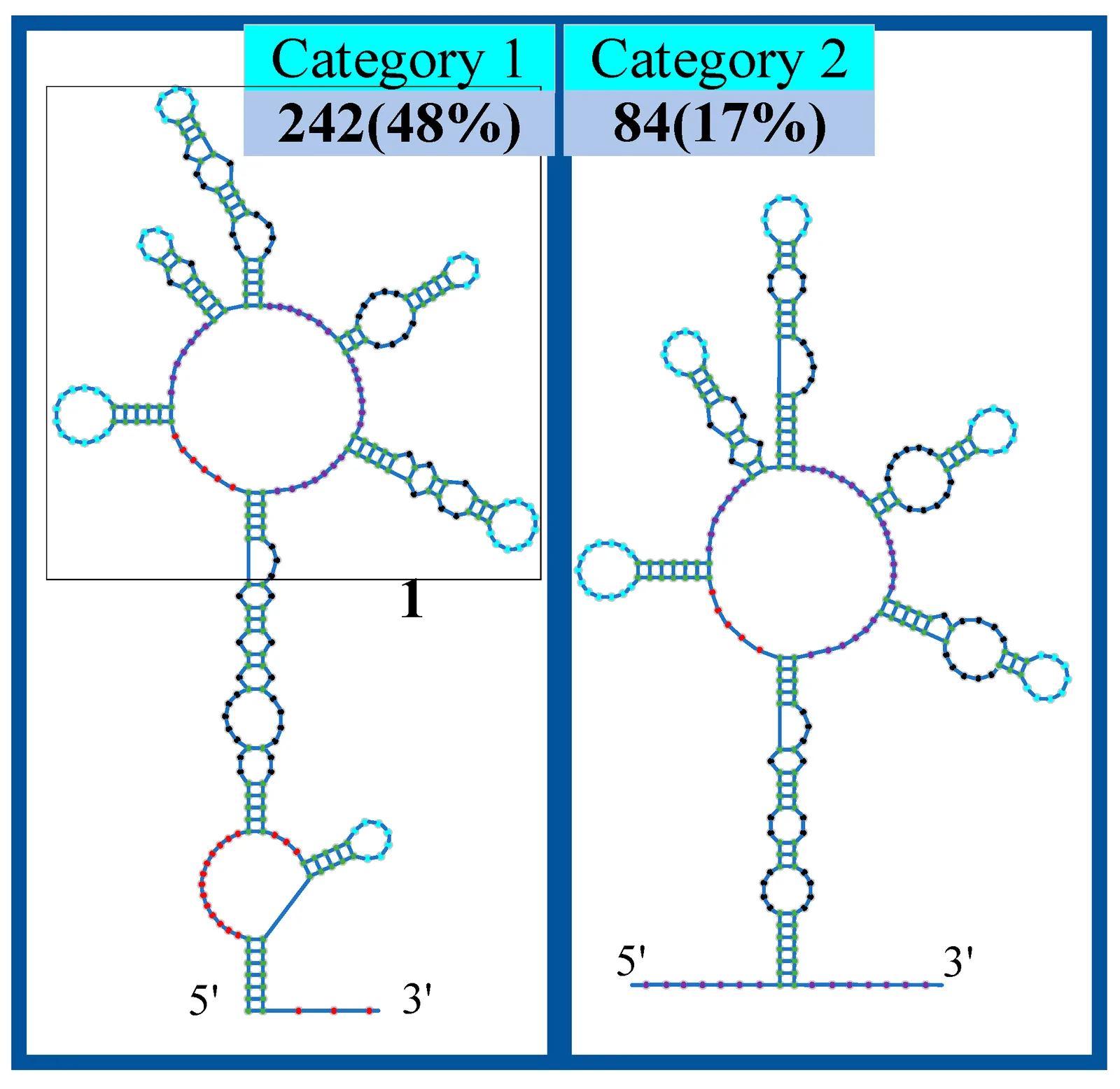
RNA secondary structures in the two categories with the most structures in the tmRNA family, as well as their quantities and proportions. The numbers in blue represent the number and proportion of structures; black, inner or convex ring; blue, hairpin ring; red, multi-branched loop or free base; green, stem area; purple, continuous unpaired sequence of at least 15 bases; unmarked, the current structure.

Most of the structures within the tmRNA family are concentrated in two categories, which together accounted for 65% of the structures in this family (Figure 5), a lower proportion than that observed for the 16S rRNA, RNase P, and SRP families. Figure 5 shows the specific structural features of these two categories, as identified by comparison with the secondary structures of the remaining, smaller categories within the tmRNA family. The RNA secondary structures of these other categories are similar to those in Category 1, except that the stem regions at position 1 may differ in length and that an additional multibranch loop may form.

### 2.6. 5S rRNA and tRNA

**Figure 6:**
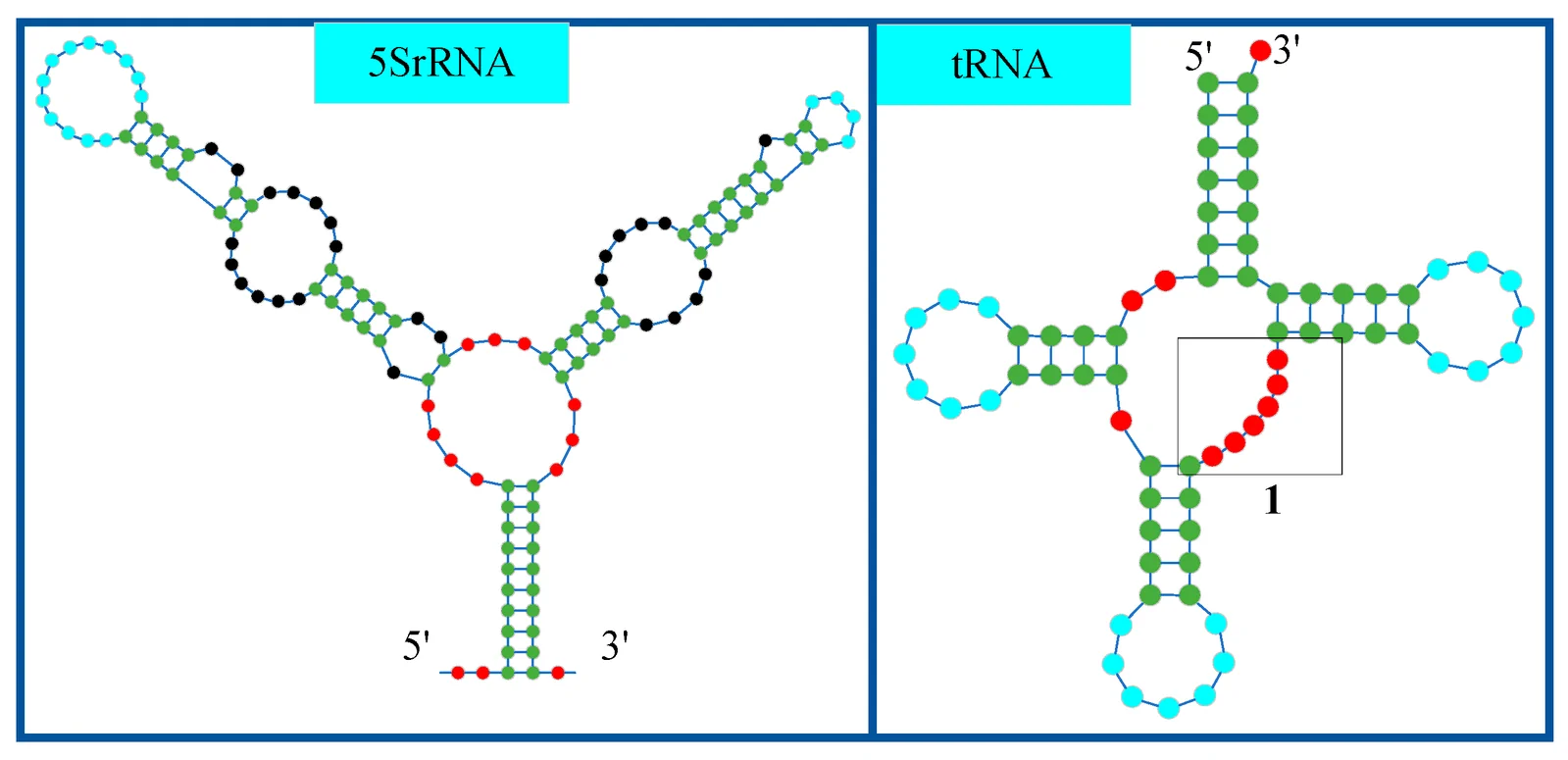
RNA secondary structures of 5S rRNA and tRNA. black, inner or convex ring; blue, hairpin ring; red, multi-branched loop or free base; green, stem area; unmarked, the current structure.

The 5S rRNA and tRNA families exhibit easily distinguishable structures: 5S rRNA displays a characteristic Y-shaped secondary structure, whereas tRNA displays the canonical cloverleaf secondary structure (Figure 6). In a small fraction of tRNA structures, position 1 forms an additional stem region.

### 2.7. Group_I_Intron

**Figure 7:**
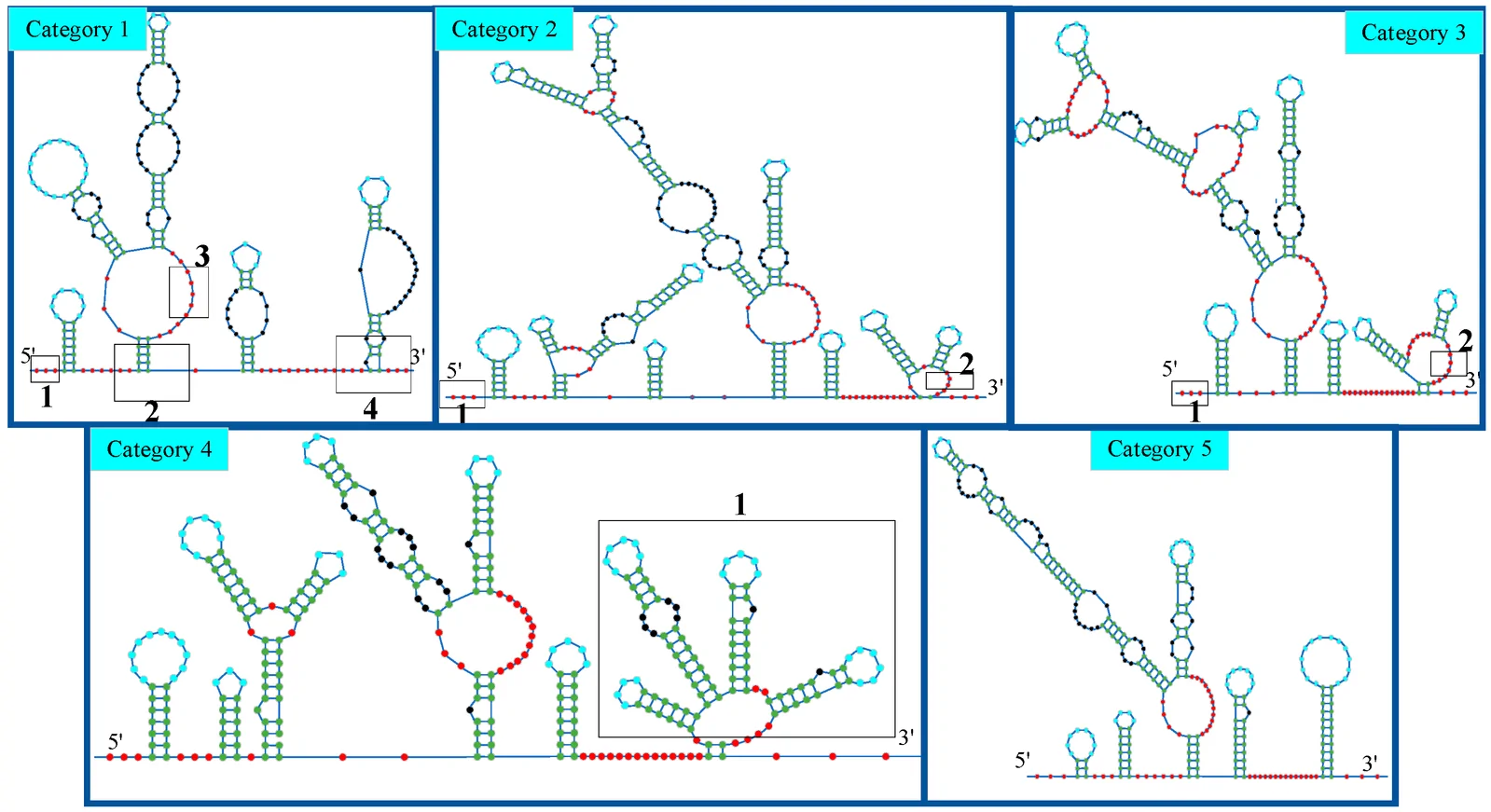
RNA secondary structures of five categories with large structural differences in the group_I_intron family.

The group_I_intron family contains multiple categories. While the number of structures in each category is roughly equal, their topologies are strongly influenced by sequence length. We selected five representative categories with substantial structural variation and length dependence and combined them with other types of RNA secondary structures to extract their features (Figure 7). In Category 1, position 1 may form an additional stem region; position 2 may form a multibranch loop with the surrounding free bases and stem region; position 3 may further give rise to a stem region; and position 4 may contain multiple surrounding stem regions, where a multibranch loop may form depending on the existing stem regions. In Category 2, position 1 may contain stem regions, depending on sequence length; position 2 may feature multiple stem regions, and its internal multibranch loop may break to form free bases. In Category 3, position 1 may contain a stem region, and position 2 may feature a stem region containing multibranch loops. In Category 4, the number of stem regions at position 1 may be reduced, and the multibranch loop may likewise break to form free bases. Categories 1 and 5 exhibit the same structural features.

### 2.8. RNA Secondary Structure Classification Based on Tree-Structure Alignment

#### 2.8.1. Distribution of Datasets

**Figure 8:**
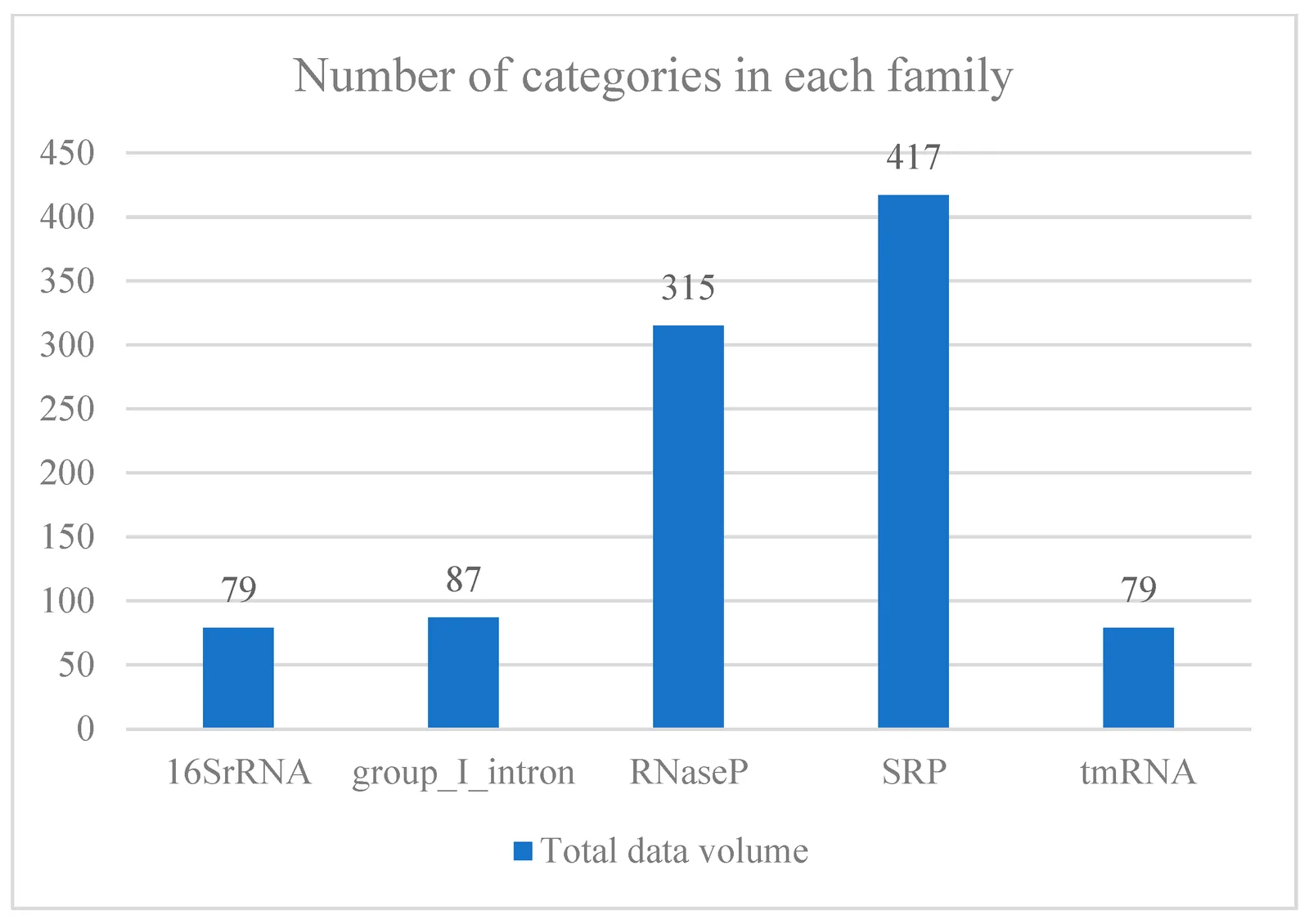
Number of structures in each family in the ArchiveII test dataset.

**Figure 9:**
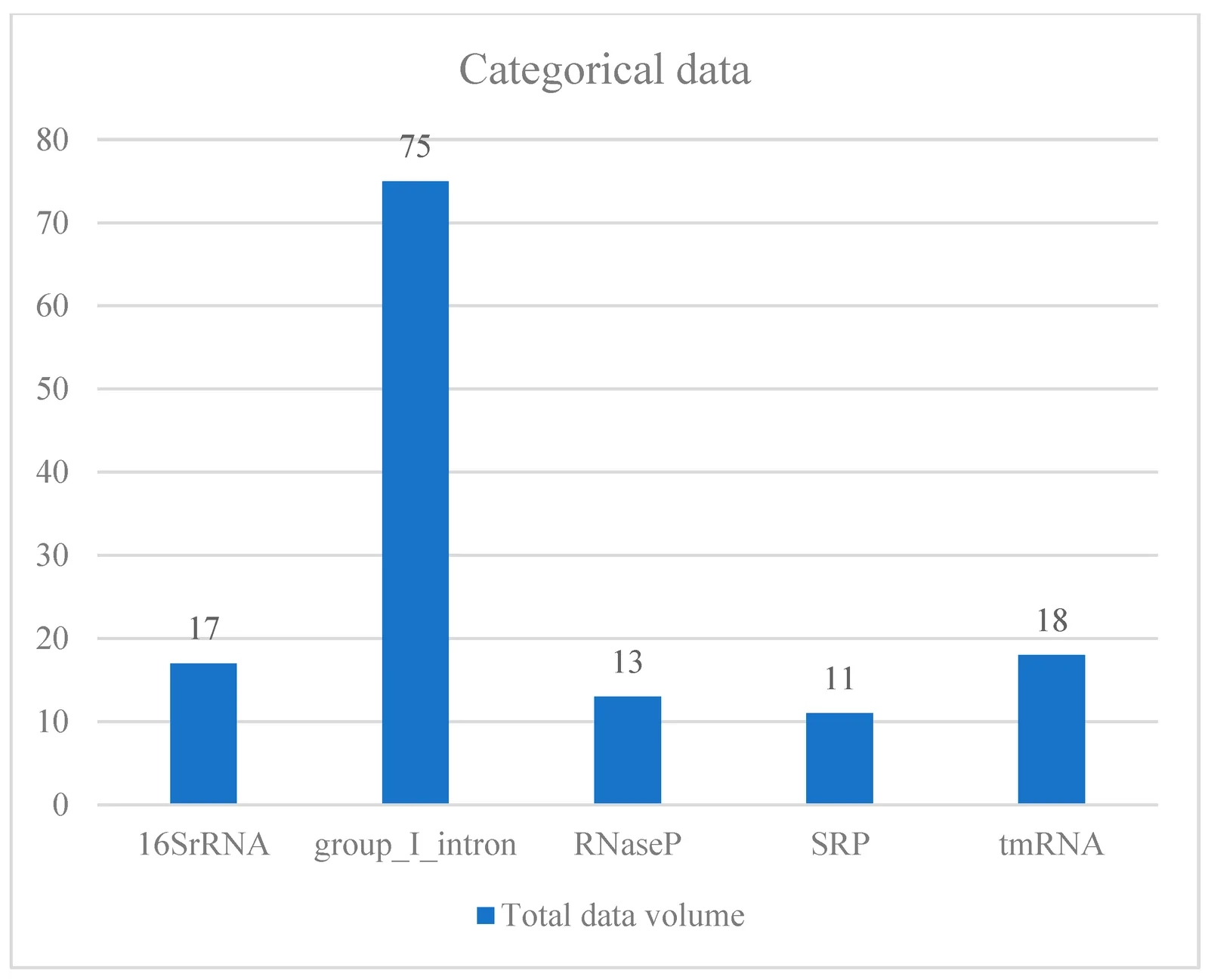
Number of categories for classification in each family.

The 5S rRNA and tRNA families were excluded from the evaluation because their structures are easily identifiable. Therefore, 977 structures were retained, and any pseudoknots in the remaining data were removed. Figure 8 presents the distribution of the remaining structures in ArchiveII. To construct the classification dataset for this family, based on the clustering results for each family, we removed classes containing fewer than four sequences, as this was judged to be the minimum number needed to establish a reliable representative structure. From the remaining classes, we selected the most frequently occurring structure within each class as the representative structure for classification. Figure 9 presents the number of categories for each family.

#### 2.8.2. RNA Secondary Structure Classification Process

**Figure 10:**
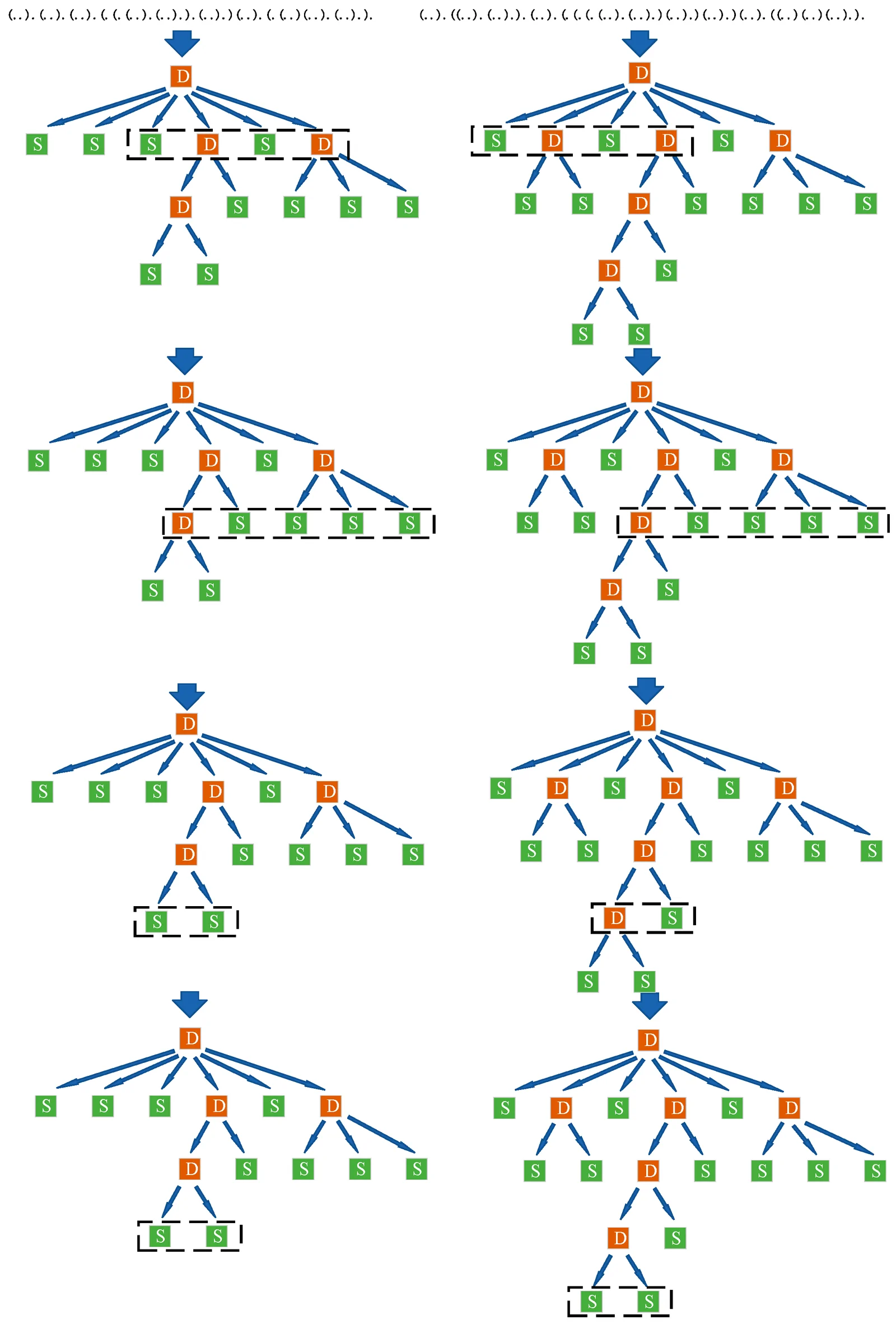
Tree-structure comparison process.

**Figure 11:**
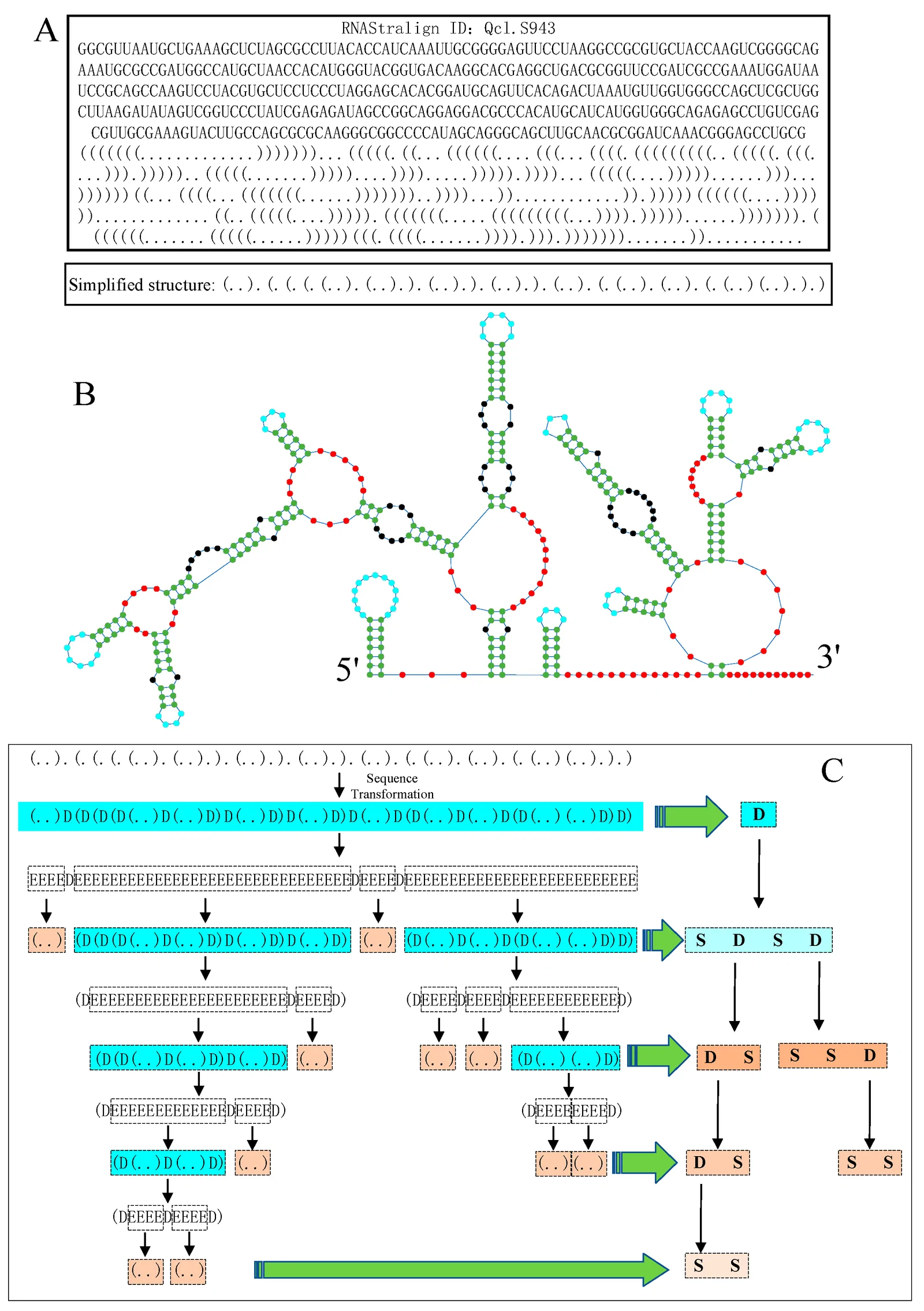
Tree construction process. (**A**) RNA sequences, dot–bracket structures, and simplified structures. (**B**) RNA secondary structure. (**C**) Tree construction process.

Each test sequence was compared with each classification sequence, and the structures of the two sequences were represented as tree structures (Figure 10). To ensure that the structure of the test sequence was highly similar to that of the classification sequence, we compared the two structures layer by layer from top to bottom according to the constructed tree structure. Figure 11 illustrates the comparison performed using the test data. We determined structural similarity using a comparison score. We evaluated identical portions of each layer node when comparing positions, employing the position-comparison process (part of the clustering process) to compare positions. In contrast to the node comparison process applied during clustering, we set different restrictions on the positional difference (*diff*) of the overall comparison node, the current positional difference (*N_diff*) of the other comparison nodes except for the first comparison node, and the length difference in the stem region (*length*1 − *length*2), with Equation (1) defining the specific limits. These thresholds (*diff* ≤ 150, |*length*1 − *length*2| ≤ 100, *N_diff* ≤ 50) were set more permissively than the clustering thresholds (*diff* ≤ 100, |*length*1 − *length*2| ≤ 20, *N_diff* ≤ 30) to account for the greater expected structural variability between an individual test sequence and a single representative structural category. These values were fixed prior to evaluation on the ArchiveII test set and were not tuned using test-set performance. Based on the proportions of successfully matched public nodes in each layer, we selected the smaller proportion as the score and then performed layer deletion according to the score, as described in Equation (2).

(1) $$\left\{\begin{array}{c} diff\leq 150,\\ \left|length1-length2\right|\leq 100,\\ N\_diff\leq 50, \end{array}\right.$$

(2) $$\left\{\begin{array}{cc} del\left(p1\to children\right), & score\leq 0.4\;and\;layer1>layer2\\ del\left(p2\to children\right), & score\leq 0.4\;and\;layer2>layer1\\ del\left(p1\to children,p2\to children\right), & score>0.4\;or\;layer1=layer2 \end{array}\right.$$

where *del* is the deletion operation; *score* is the comparison score; *layer*1 is the number of layers in the test tree; and *layer*2 is the number of layers in the feature classification tree. If the two-layer node structures were identical, 0.5 points were added.

We aggregated the comparison scores of each layer to obtain the total comparison score of the current test tree. Based on the comparison scores between the current test sequence and each classification sequence, we assigned the test sequence to the family corresponding to the classification sequence with the highest comparison score. If there was more than one highest alignment score, we selected the family corresponding to the classification sequence that required the fewest comparisons with the current test sequence.

#### 2.8.3. Classification Results and Analysis

**Figure 12:**
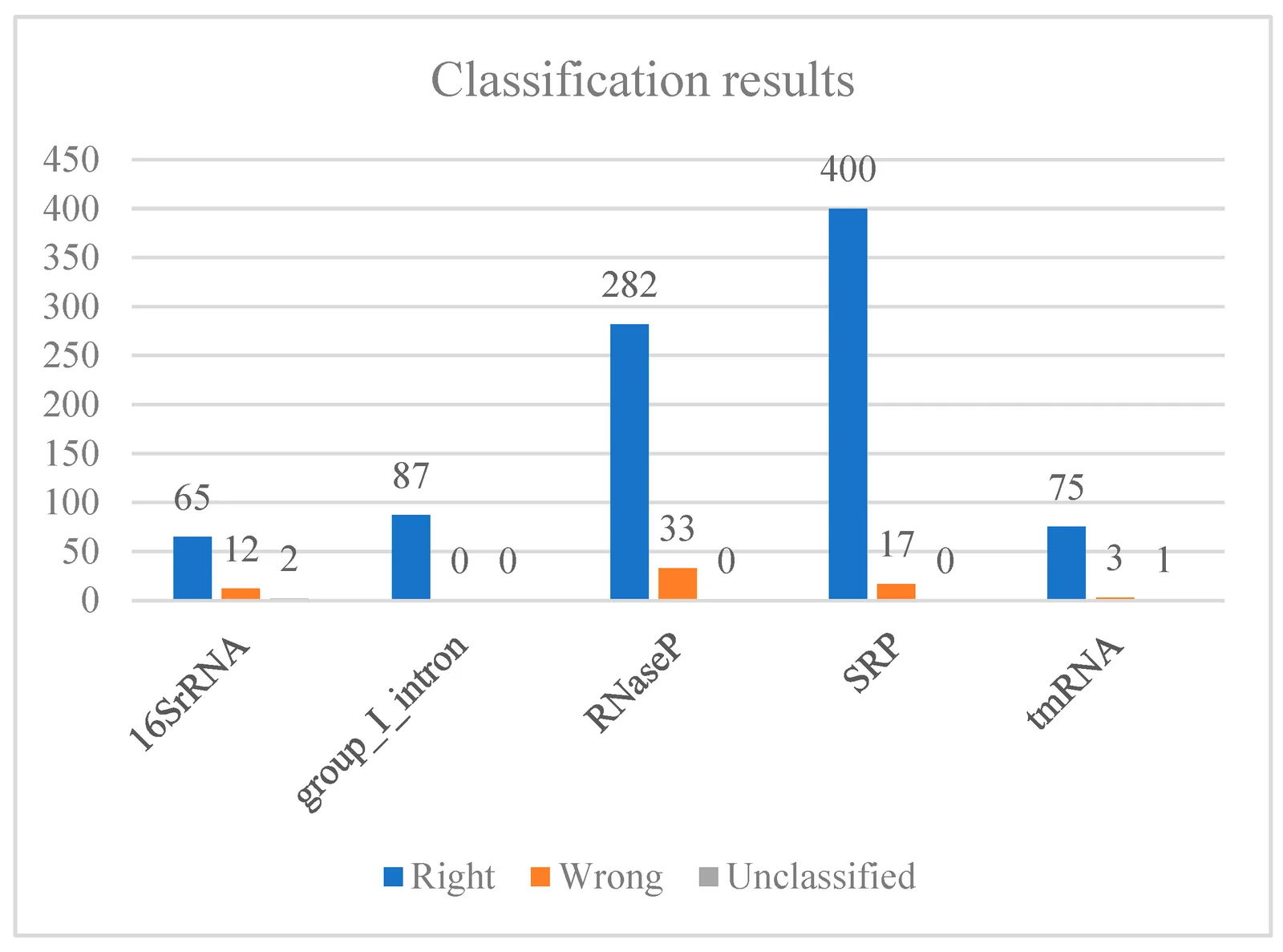
Classification results.

Figure 12 presents the classification results. Owing to the small amount of data in the “unknown” category, sequences that did not sufficiently match any representative structure under our comparison criteria were excluded from further analysis (affecting three sequences). Table 1 presents the confusion matrix of the classification results, describing the extent of misclassification for each family. To examine classification accuracy by family, we calculated the numbers of true positives (*TP*), true negatives (*TN*), false positives (*FP*), and false negatives (*FN*) (Table 2).

To examine algorithm performance by family, we calculated precision (the ratio of *TP*s to the sum of *TP*s and *FP*s), recall (the ratio of *TP*s to the sum of *TP*s and *FN*s), and the *F*1-score (the harmonic mean of precision and recall) using Equations (3)–(5) (Table 3). The algorithm exhibited the worst performance for the 16S rRNA family, indicating that other families were more likely to be misclassified as 16S rRNA.

(3) $$Precision=\frac{TP}{TP+FP}$$

(4) $$Recall=\frac{TP}{TP+FN}$$

(5) $$F1=\frac{2\times Precision\times Recall}{Precision+Recall}$$

To examine the overall algorithm performance across all families, we calculated micro-average metrics using *Precision_micro_* (to evaluate precision across all families), *Recall_micro_* (to evaluate recall across all families), and *F*1*_micro_* (to evaluate the harmonic mean across all families), as per Equations (6)–(8):

(6) $${Precision}_{micro}=\frac{\sum_{i=1}^{N}{TP}_{i}}{\sum_{i=1}^{N}\left({TP}_{i}\;+\;{FP}_{i}\right)}$$

(7) $${Recall}_{micro}=\frac{\sum_{i=1}^{N}{TP}_{i}}{\sum_{i=1}^{N}\left({TP}_{i}+{FN}_{i}\right)}$$

(8) $${F1}_{micro}=\frac{2\times {Precision}_{micro}\times {Recall}_{micro}}{{Precision}_{micro}+{Recall}_{micro}}$$

where *N* is the total number of categories, and *i* denotes the corresponding family. Notably, the overall algorithm performance in all families was good (Table 4).

To eliminate errors due to the uneven distribution of data across the families, we calculated macro-average metrics using *Precision_macro_* (average precision across all families), *Recall_macro_* (average recall across all families), and *F*1*_macro_* (the average harmonic mean across all families), as per Equations (9)–(11).

(9) $${Precision}_{macro}=\frac{1}{N}\sum_{i=1}^{N}\frac{{TP}_{i}}{{TP}_{i}+{FP}_{i}}$$

(10) $${Recall}_{macro}=\frac{1}{N}\sum_{i=1}^{N}\frac{{TP}_{i}}{{TP}_{i}+{FN}_{i}}$$

(11) $${F1}_{macro}=\frac{1}{N}\sum_{i=1}^{N}\frac{2\times {Precision}_{i}\times {Recall}_{i}}{{Precision}_{i}+{Recall}_{i}}$$

Given that the classification accuracy for the 16S rRNA family was low, resulting in a high rate of structures being misclassified as 16S rRNA, the resulting macro-average was also slightly low. However, the overall model performance remained good (Table 4). Equation (12) weights raw *TP* counts rather than per-class precision (*TP*/(*TP* + *FP*)). Equation (13) weights raw *FP* counts, which do not correspond to any standard definition of recall (which should be based on *TP* and *FN*, not *FP*).

To prevent extreme variations in data volume (too much or too little) across different RNA families, we evaluated the weighted averages for each metric. Accordingly, *Precision_weight_* (the sum of the weighted precision of each family), *Recall_weight_* (the sum of the weighted recall of each family), and *F*1*_weight_* (the sum of the weighted harmonic mean of each family) were computed as per Equations (12)–(14):

(12) $${Precision}_{weight}=\sum_{i=1}^{N}\frac{TP_{i}}{TP_{i}+FP_{i}}\times \frac{count_{i}}{AllCount}$$

(13) $${Recall}_{weight}=\sum_{i=1}^{N}\frac{TP_{i}}{TP_{i}+FN_{i}}\times \frac{count_{i}}{AllCount}$$

(14) $${F1}_{weight}=\sum_{i=1}^{N}{F1}_{i}\times \frac{{count}_{i}}{AllCount}$$

where *AllCount* is the total volume of data in all families and ${count}_{i}$ is the data volume of family *i* (Table 4). These results revealed good overall performance, indicating that the proposed algorithm is not substantially affected by families with excessive or limited data. In our tests, the empirical runtime of our algorithm scaled favorably with sequence length, with time complexity being approximately O(*n*). A rigorous asymptotic complexity analysis and a head-to-head runtime comparison with tools such as bpRNA-align were beyond the scope of the present study and represent an important direction for future validation.

#### 2.8.4. Stem Repair Based on RNA Secondary Structure Classification

**Figure 13:**
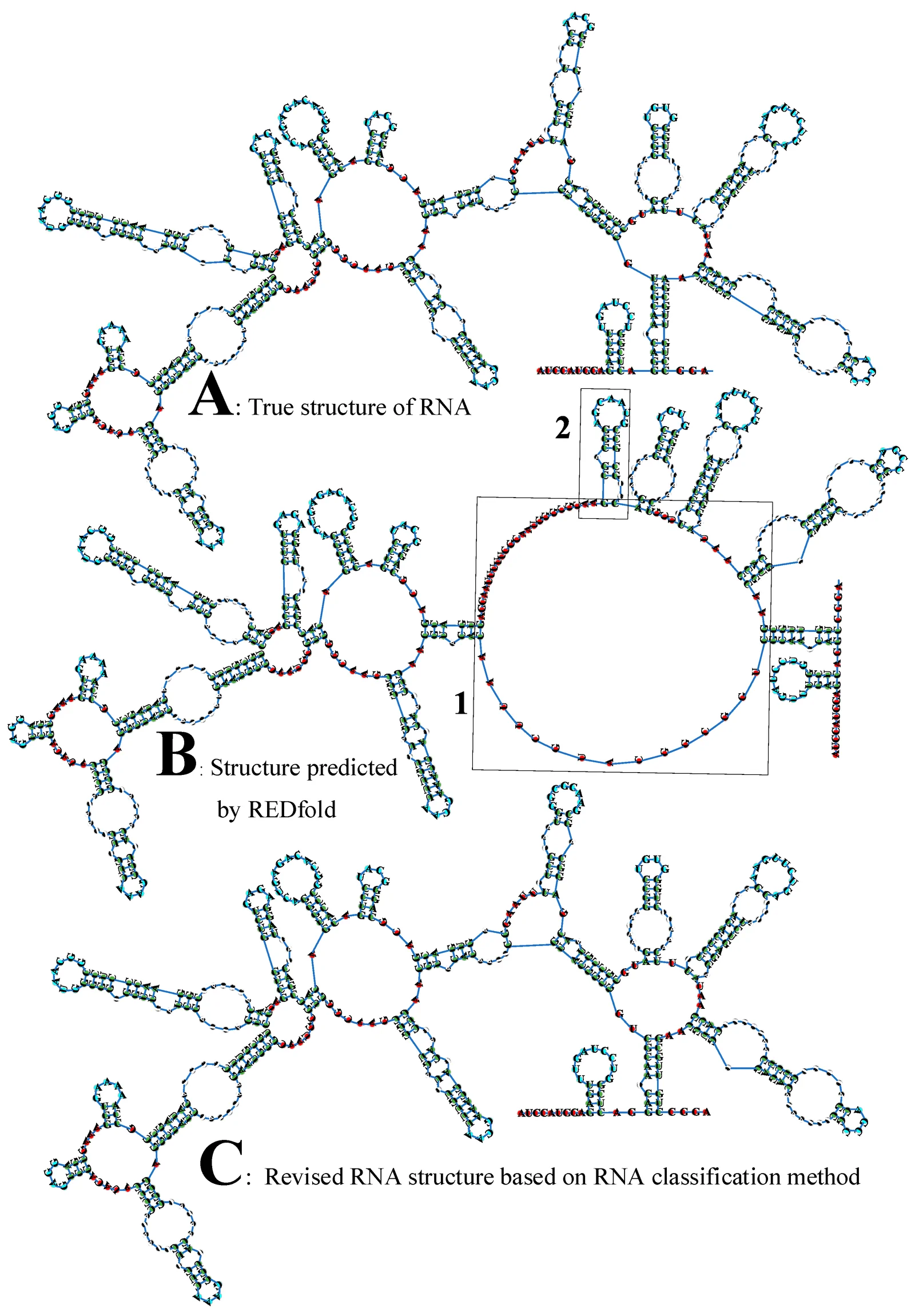
RNA structure modification.

To demonstrate the practical application of our algorithm, we used REDfold [24] to predict the RNA secondary structure of the ArchiveII sequence 16sC.reinhardtii.chloro_domain1 and repaired its stem region based on the predicted results. Figure 13A presents the true structure, and Figure 13B presents the predicted secondary structure. We transformed the REDfold-predicted secondary structure into a tree structure and selected the appropriate structural category using our tree-structure comparison method. Subsequently, we partially repaired the REDfold-predicted secondary structure according to the selected category; based on the classification results, its framework was similar to that of 16S rRNA Category 2 (Figure 2). We then compared the predicted structure with the classified structure by constructing a tree structure; each stem region in the predicted structure was compared with its corresponding stem region in the classified structure based on tree-structure alignment. If a stem region in the predicted structure did not appear in the classification structure, it was generated according to its relative position in the classification structure, and any conflicting surrounding base-pairing was canceled. Specifically, the bases of the multi-branched loop at position 1 (Figure 13) were paired according to the corresponding position in the Category 2 structure (Figure 2), and the stem region at position 2 was regenerated according to the corresponding position of the stem region in the Category 2 structure. We used the *F*1 score for RNA secondary structure prediction as the evaluation metric. The *F*1 score before repair was approximately 0.7, which improved to over 0.8 after repair. Compared with the unmodified structure, the modified RNA structure (Figure 13C) was more similar to the actual structure, demonstrating the effectiveness of this method. Notably, this method can also modify RNA structures predicted by other algorithms, thereby assisting in RNA structure prediction and family classification.

## 3. Discussion

In this study, we present Tree_RNA-Align, an RNA secondary structure clustering and classification algorithm based on tree-structure alignment. First, RNA secondary structures from the 16S rRNA, group_I_intron, RNase P, SRP, tmRNA, 5S rRNA, and tRNA families were clustered separately. Prior to clustering, multibranch loops within each RNA secondary structure were identified, and the stem regions were segmented according to the positions of the multi-branch loops to construct a tree structure. During clustering, two tree structures were compared in a hierarchical manner from bottom to top, following a tree-like structure, and the RNA structures that met the criteria were grouped into categories. Based on the clustering results, the potential structures within each family were identified, and the appropriate representative structure was selected to construct the classification dataset. For classification, the test RNA sequence was compared with the representative structure by constructing a tree structure from top to bottom and assigning appropriate scores to accurately and rapidly classify the RNA secondary structure.

We evaluated the performance of this algorithm for each family. This algorithm exhibited relatively lower classification accuracy for 16S rRNA structures but showed stronger performance for group_I_intron, RNase P, SRP, and tmRNA. The reason for the poor performance of the 16S rRNA family in classification is shown in the confusion matrix in Table 1. Specifically, we found that when classifying the remaining four families, many structures were misclassified as 16S rRNA. This indicates that some classes in the remaining four families may share tree structure similarities with certain classes in the 16S rRNA family. From a structural variation perspective, we found differences in the number of base pairs in each stem region within the clustering results; however, in the overall structure, there were some differences in the internal base pairs based on the specific structure. To further evaluate the algorithm’s performance, we calculated micro-, macro-, and weighted-average metrics and found that it achieved high prediction accuracy, making it suitable for RNA secondary structure classification. Finally, we demonstrated the algorithm’s capability to repair an RNA secondary structure.

However, the algorithm has certain limitations. Considering that Tree_RNA-Align compares entire stem regions as a whole, it may not be suitable for highly detailed structural comparisons. The clustering component of our algorithm effectively clusters structures within known families. However, for RNA sequences from unknown families, we recommend using a different classification algorithm prior to clustering. Moreover, because our clustering algorithm is executed in the order of the records within each dataset, the number of clusters generated may differ if the order of the records changes. Nevertheless, extensive testing indicated that sequence order had a minimal effect on clustering. Using 16S rRNA as an example, across 20 trials with randomly permuted input orders, we observed that the cluster variance was only 0.45, indicating that the input sequence order had a minimal effect on the resulting cluster assignments for these families.

## 4. Methods

### 4.1. Dataset Selection and Processing

**Figure 14:**
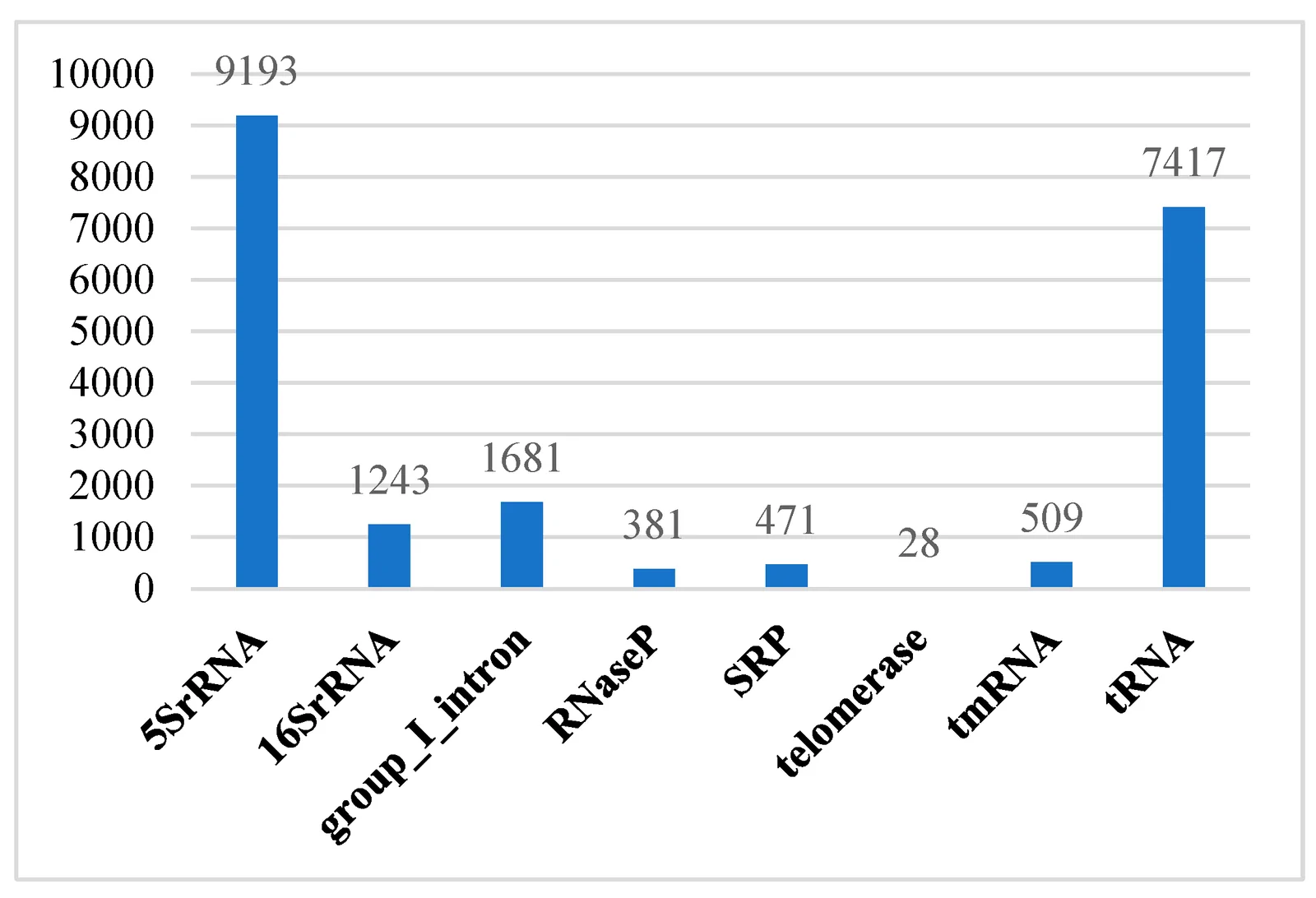
Sequence distribution in the RNAStralign dataset by structural family following data processing and sequence selection.

To train and evaluate this method, we used the RNAStralign [25] dataset, which contains 37,149 sequences. This dataset comprises structures from eight structural families: 5S rRNA, 16S rRNA, group_I_intron, RNase P, SRP, telomerase, tmRNA, and tRNA. Using the data processing protocol described in MXfold2 [26], we removed redundant sequences, sequences longer than 600 bases, and pseudoknots, resulting in a final dataset of 20,923 sequences. Figure 14 presents the distribution of the sequences included in the clustering analysis. As the telomerase family contained only 37 sequences, which is insufficient for reliable within-family clustering given the diversity of structures typically observed, it was excluded from further analysis. Table 5 summarizes the final composition of the RNAStralign dataset; the majority of the RNA families contain sequences shorter than 600 bases. As sequences longer than 600 bases are typically concentrated in the 16S rRNA family, they were consistently removed.

For subsequent classification operations, we used the ArchiveII dataset as the independent test set [27]. We removed duplicate sequences from the training set, and any pseudoknots in the remaining data were removed. Subsequent dataset processing will be further processed according to the clustering results.

### 4.2. RNA Secondary Structure Conversion

#### 4.2.1. Sequence Conversion

We use dots and brackets to represent the secondary structure of RNA. The dot represents an unpaired free base, while the left and right brackets represent the corresponding paired base. Each paired base must meet the “AU”, “GC”, and “UG” pairing relationship.

**Figure 15:**
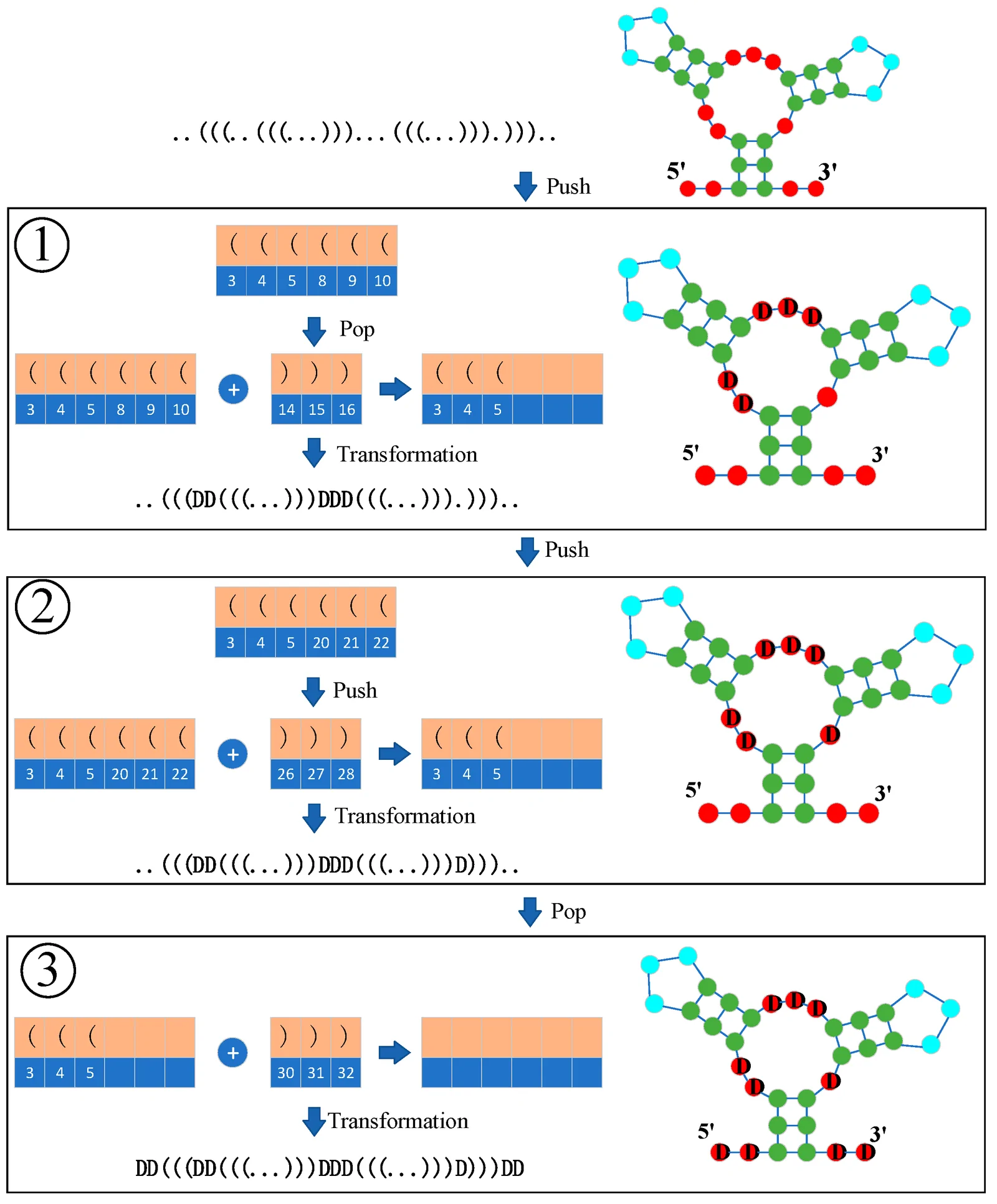
Sequence conversion process.

Using a stack-based approach, multibranch loops are represented using the letter “D.” This procedure specifically targets junctions at which one helix terminates and a new helix begins, distinguishing multibranch loops from internal loops and bulge loops, which occur within a single helical stem rather than between two helices. The following steps are performed: As the RNA secondary structure in dot–bracket notation is traversed, each opening bracket “(“ is placed on the stack, and its position is recorded. When a closing bracket “)” is reached, the corresponding opening bracket is removed from the stack, and the positions of both the opening and closing brackets are recorded. When the next opening bracket is encountered after a closing bracket, all dots between the corresponding opening bracket (to the left) and closing bracket are changed to “D.” The next closing bracket to the right is then traversed and processed in the same manner. If there are no dots between the brackets, no mark is made; however, the positions of the corresponding closing and opening brackets are still recorded as the boundaries of the multibranch loop. This process is repeated until the entire sequence is traversed and converted (Figure 15).

#### 4.2.2. Construction of Tree Structure

**Figure 16:**
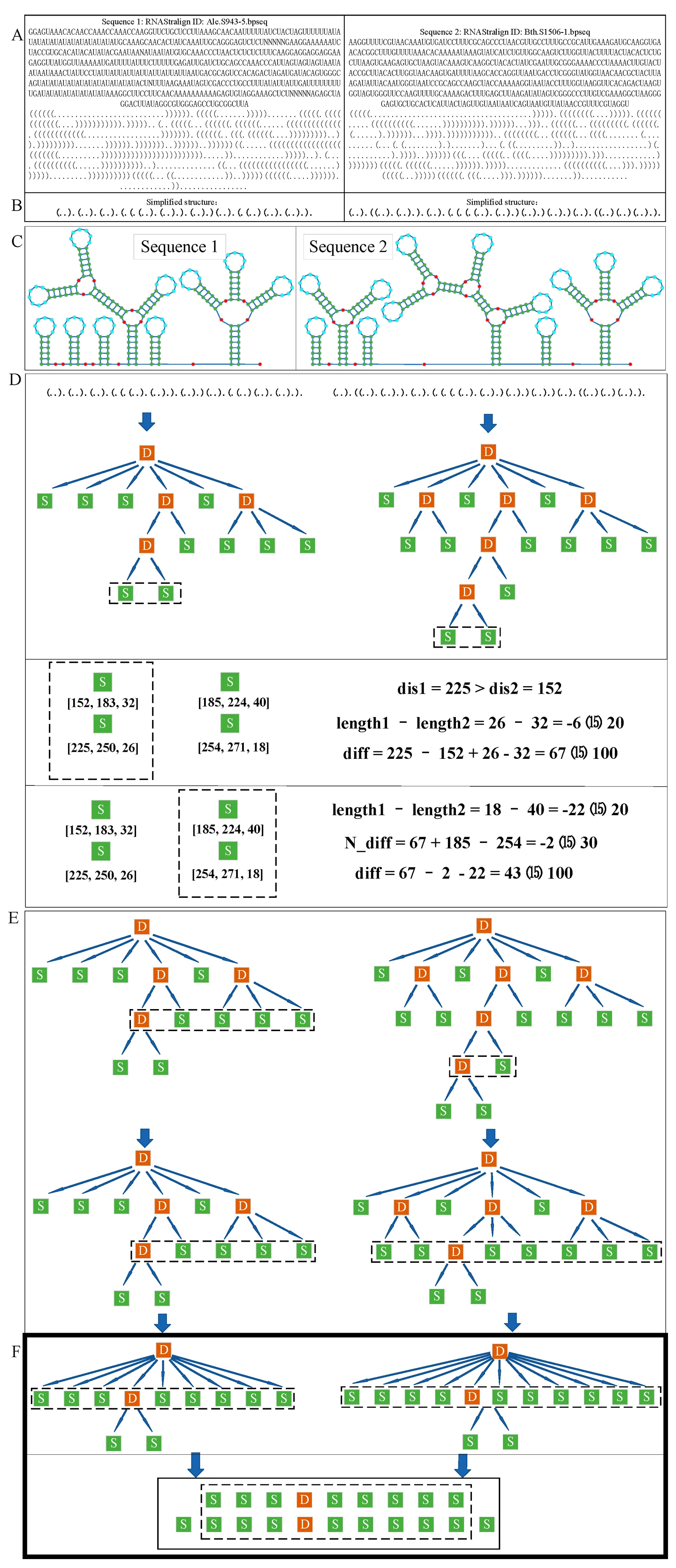
Process of comparing two RNA secondary structures. (**A**) Sequence and dot–bracket structure of the two RNAs. (**B**) Simplified two-point bracket structure. (**C**) Visualization of the two RNA structures. (**D**) Specific node comparison process. (**E**) Tree alignment and node merging. (**F**) Extraction of common child nodes.

The RNA secondary structure is segmented into stem regions according to the intervals of the multibranch loops. After sequence conversion, each substructure of the RNA secondary structure, except for the multi-branch loops, is recursively converted into multi-branch loop–stem region structures using the same procedure. Within each resulting substructure, each subregion of the current stem region is segmented based on its multi-branch loop, recorded, and used as a subnode. If the current substructure contains multi-branch loops, it is denoted as “D”; if not, it is denoted as “S”. For example, given a stem region X containing a multibranch loop that separates it into sub-stems X1 and X2, X is labeled “D” and becomes the parent node; X1 and X2 are its children, each subsequently labeled “S” or “D” depending on whether they themselves contain a multi-branch loop. The start and end positions and length of the segmented stem region are recorded, and the start and end positions of each stem subregion in the current stem are denoted as “E”. If the segmented stem subregion contains multi-branched loops, this node is denoted as “D”; if not, it is denoted as “S”. The “D” nodes among the children are then treated as parent nodes, and the steps are repeated until all child nodes are resolved to “S”. Figure 16 presents the tree construction process after simplification of the secondary structure.

#### 4.2.3. RNA Secondary Structure Clustering

RNA secondary structures are then clustered within each RNA family. The first unclustered RNA secondary structure is selected and compared with all remaining structures using their tree representations. Subsequently, all child nodes within the same parent node are compared. Nodes are compared based on the conditions specified in Equation (15). Equation (17) defines the current positional difference between all nodes being compared except the first comparison node. As the processing methods for the first node and the remaining nodes differ, we utilize Equations (16) and (18); here, Equation (16) represents the difference in position for the first comparison node, while Equation (18) represents the difference in position for all subsequent nodes. To set the criteria for the three variables in Equation (15), RNA secondary structures sharing identical tree structures in each family are clustered, and the largest group from each family is chosen for analysis. For the selected groups, we calculate the maximum value for each variable using Equations (16)–(18). We then further relax the restrictions to obtain. the final required constraints.

(15) $$\left\{\begin{array}{c} diff\leq 100,\\ \left|length1-length2\right|\leq 20,\\ N\_diff\leq 30, \end{array}\right.$$

(16) $$diff=\left\{\begin{array}{c} dis1-dis2+length1-length2,\;\;dis1\geq dis2\\ dis2-dis1+length2-length1,\;\;dis2>dis1 \end{array}\right.$$

(17) $$N\_diff=\left\{\begin{array}{c} diff+N\_dis2-N\_dis1,\;\;dis1\geq dis2\\ diff+N\_dis1-N\_dis2,\;\;dis2\geq dis1 \end{array}\right.$$

(18) $$diff=\left\{\begin{array}{c} diff+N\_diff+length1-length2,\;\;dis1\geq dis2\\ diff+N\_diff+length2-length1,\;\;dis2>dis1 \end{array}\right.$$

where *diff* is the positional difference between the nodes being compared, *N_diff* is the current positional difference in the other comparison nodes except the first comparison node, *dis*1 and *dis*2 are the starting positions of the first comparison node, *N_dis*1 and *N_dis*2 are the starting positions of the other comparison nodes, and *length*1 and *length*2 are the lengths of the two comparison nodes.

The alignment of two representative RNA structures is presented in Figure 16A–C. Figure 16D illustrates the bottom-node comparison process according to Equations (1)–(3), in which the first element in the array is the starting position of the node, the second element is the end position of the node, and the third element is the length of the node. The process of merging the secondary structure nodes of the two sequences is presented in Figure 16E,F. The comparison is performed from the bottom node to the top node, according to the tree’s hierarchical structure. During the bottom-node comparison, sequences are skipped if the structures differ from the alignment or do not meet the alignment conditions, and the comparison proceeds to the next sequence. If the bottom nodes are the same and meet the comparison conditions, the nodes of the previous layer are compared. If the nodes of the previous layer are the same, the comparison continues up the tree, and the nodes are compared until the second layer of nodes is reached. If the nodes of the previous layer are different, a node-merging operation is performed. Equation (19) describes the specific operation of node merging, whereby the parent node of the current node replaces the current node and is combined and compared with other nodes of the current layer:

(19) $$\left\{\begin{array}{c} s1\to parent=s1,\;\;s1\neq s2,layer1\geq layer2\\ s2\to parent=s2,\;\;s1\neq s2,layer2>layer1 \end{array}\right.$$

where *s*1 and *s*2 are the two-layer nodes to be compared, *parent* refers to the parent node of the current layer node, and *layer*1 and *layer*2 represent the corresponding layers of the two-layer nodes to be compared.

The number of clusters in the other three families was significantly affected by the threshold, whereas the number of clusters in 16S rRNA and tmRNA was less affected by the threshold. In these three families, when the threshold was less than 50%, the number of clusters remained relatively stable; however, when the threshold exceeded 50%, the number of clusters increased relatively (Table 6). To ensure the stability of the resulting clusters, we selected 50% as the threshold.

Clustering is established when the proportion of matched nodes between the two trees exceeds 50% of the total number of substructures. This threshold was selected empirically [state basis]; a sensitivity analysis across a range of thresholds (e.g., 30–70%) is provided in the Supplementary Materials to demonstrate the robustness of the resulting cluster assignments. Figure 16F illustrates the node-extraction process.

Upon completion of the tree-structure comparison between the currently selected RNA secondary structure and all other remaining structures, the first non-clustered RNA secondary structure is selected, proceeding sequentially according to the dataset order. A subsequent round of tree-structure comparison is then conducted with the remaining non-clustered RNA secondary structures. This procedure is repeated until all RNA secondary structures have been successfully clustered.

## 5. Conclusions

This study introduces Tree_RNA-Align, a tree-based approach that enables both clustering and classification of RNA secondary structures by preserving the hierarchical relationships among stems and multibranch loops. When applied to seven RNA families, the clustering method resolved structurally distinct categories within each family, including multiple well-separated subtypes in 16S rRNA, RNase P, and SRP. Classification using the same tree-alignment framework achieved a micro-averaged *F*1 score of 0.933 across five families, with the strongest per-family performance observed for tmRNA (*F*1 = 0.981) and the weakest for 16S rRNA (*F*1 = 0.760). A proof-of-concept demonstration showed that classification-guided repair of an algorithmically predicted 16S rRNA structure yielded a conformation more consistent with the known reference, suggesting that classification results could inform secondary structure prediction pipelines. Future work should include quantitative benchmarking against established RNA alignment and clustering tools, formal characterization of parameter sensitivity, and extension to datasets containing pseudoknots and longer RNAs.

## Figures and Tables

**Table 1:** Confusion matrix of the classification results.

	16SrRNA	Group_I_Intron	RNaseP	SRP	tmRNA
16SrRNA	65	3	4	5	0
group_I_intron	0	87	0	0	0
RNaseP	14	7	282	12	0
SRP	13	0	4	400	0
tmRNA	2	0	0	1	75

**Table 2:** True- or false-positive and true- or false-negative values for each analyzed family.

	*TP*	*FP*	*TN*	*FN*
16SrRNA	65	29	868	12
group_I_intron	87	10	877	0
RNaseP	282	8	651	33
SRP	400	18	539	17
tmRNA	75	0	896	3

*TP*, true positive; *FP*, false positive; *TN*, true negative; *FN*, false negative.

**Table 3:** Performance results for each analyzed family.

	*Precision*	*Recall*	*F*1
16SrRNA	0.691	0.844	0.760
group_I_intron	0.897	1.000	0.946
RNaseP	0.972	0.895	0.932
SRP	0.957	0.959	0.958
tmRNA	1.000	0.962	0.981

**Table 4:** Algorithm performance based on average effects.

	*Precision*	*Recall*	*F*1
Micro	0.933	0.933	0.933
Macro	0.903	0.932	0.915
Weight	0.939	0.933	0.935

**Table 5:** Summary of RNA structural families in the RNAStralign dataset.

RNA Type	Length (Bases)	Total Number of Sequences
16SrRNA	54–1851	12,608
group_I_intron	163–615	2135
SRP	30–553	601
RNaseP	189–486	467
tmRNA	102–437	637
telomerase	382–559	37
5SrRNA	104–132	11,419
tRNA	59–95	9245
Total	30–1851	37,149

**Table 6:** Influence of different thresholds on the number of clusters.

	30%	40%	50%	60%	70%
16S rRNA	59	59	60	60	61
group_I_intron	139	149	155	173	184
RNase P	24	24	26	31	34
SRP	20	22	22	34	38
tmRNA	45	45	46	46	46

## Data Availability

The original data presented in the study are openly available in https://github.com/Xu-Chengzhen/Tree_RNA-Align (accessed on 10 August 2026).

## Supplementary Materials

https://github.com/Xu-Chengzhen/Tree_RNA-Align (accessed on 11 August 2026).

## Abbreviations

The following abbreviations are used in this manuscript:

*diff*	positional difference
*F*1*_macro_*	average of the harmonic means across all families
*F*1*_micro_*	micro-average to evaluate the harmonic mean in all families
*F*1*_weight_*	sum of the weighted harmonic mean of each family
*FN*	false negative
*FP*	false positive
*N_diff*	current positional difference
ncRNA	non-coding ribonucleic acid
PDB	Protein Data Bank
*Precision_macro_*	average precision in all families
*Precision_micro_*	micro-average of precision in all families
*Precision_weight_*	sum of the weighted precision of each family
*Recall_macro_*	average recall in all families
*Recall_micro_*	micro-average of the evaluation recall in all families
*Recall_weight_*	sum of the weighted recall of each family
RNA	ribonucleic acid
*TN*	true negative
*TP*	true positive
